# Low and High Ankle-Brachial Index Are Both Associated with Mortality in German Nursing Home Residents—The Five-Year Follow-Up of the “Allo-Study” Cohort

**DOI:** 10.3390/jcm12134411

**Published:** 2023-06-30

**Authors:** Anna Dorn, Bernhard Dorweiler, Wael Ahmad, Spyridon Mylonas, Ingrid Becker, Payman Majd

**Affiliations:** 1 Vascular Surgery Department, Protestant Hospital, 51465 Bergisch Gladbach, Germany; p.majd@evk.de; 2 Vascular Surgery Department, University Hospital of Cologne, 50937 Cologne, Germany; gefaesszentrum@uk-koeln.de (B.D.); wael.ahmad@uk-koeln.de (W.A.); spyridon.mylonas@uk-koeln.de (S.M.); 3 Institute of Medical Statistics and Computational Biology, University Hospital of Cologne, 50937 Cologne, Germany; ingrid.becker@uni-koeln.de

**Keywords:** peripheral arterial disease, ankle-brachial index, screening, epidemiology, nursing homes, aged

## Abstract

Peripheral arterial disease (PAD) is associated with high cardiovascular morbidity and mortality. We aimed to examine this relation in a population that tends to be under-represented in research on the topic. In a prospective observational cohort study, residents of 45 nursing homes in Germany were screened for pathological ankle-brachial index (ABI) and observed for five years. Of 1333 participants (median age 84 years), 55.5% had a pathological ABI (≤0.9 or >1.4) on one or both legs. 84.7% of the probands with a low ABI (indicating PAD) had no previously known PAD diagnosis. The five-year mortality was 73.0%. Mortality was higher in individuals with a pathological ABI (76.5%) than in those with a normal ABI (68.7%, *p* = 0.003). An ABI > 1.4 was associated with a higher mortality (79.4%) than a reduced (74.7%) or normal ABI (68.7%, *p* = 0.011). Pathological ABI values were associated with an increase in mortality after correction for age, sex and all recorded comorbidities, including cardiac disease. Although PAD is highly prevalent in nursing home residents, it is underdiagnosed and undertreated. In the study cohort, both high and low ABI were important predictors of mortality. PAD deserves more attention in this high-risk population.

## 1. Introduction

Global disability and mortality associated with peripheral artery disease (PAD) are increasing [1]. Predominantly, older patients are affected [2,3]. PAD has been observed to be associated with considerable cardiovascular morbidity and mortality [4,5,6]. The ankle-brachial index (ABI) is a well-established noninvasive screening method to detect PAD [7].

Epidemiological trials on the prevalence of PAD do not always include elderly, frail patients. In examinations in an ambulatory setting, such as the observational German Epidemiological Trial on Ankle Brachial Index (getABI study) [3], individuals with impaired mobility or cognitive deficits may be under-represented. However, a higher prevalence of vascular disease is likely, especially in multi-morbid patients. 

The prevalence of PAD in the “Allo-Study” cohort of nursing home residents and the one-year follow-up was published in 2019 by Galas et al. [8]. A high rate of underdiagnosis and undertreatment of PAD patients were noted. There was no statistically significant difference in the one-year survival of probands with and without PAD.

In this work, we examine the relation between PAD and the five-year mortality in this population and analyze possible influencing factors. 

## 2. Materials and Methods

### 2.1. Study Population

**Figure 1:**
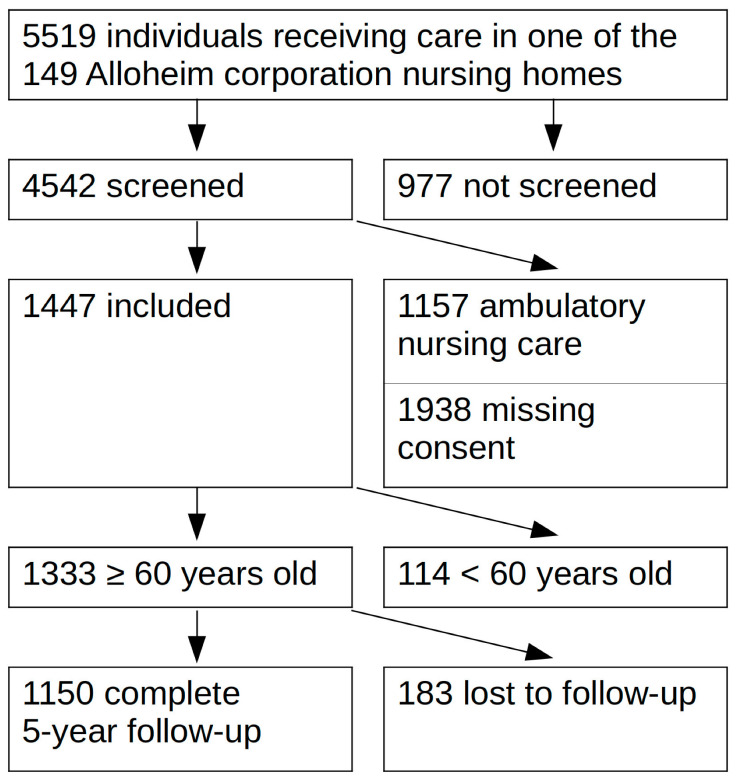
Flow chart of screening and recruitment of the Allo-Study population.

In a prospective cohort study, all individuals cared for in one of the 149 nursing homes of the “Alloheim” collaboration were screened (Figure 1). At the time of enrollment (starting in 2014), Alloheim was the third biggest private company providing inpatient nursing care in Germany.

### 2.2. Inclusion and Exclusion Criteria

The study recruited all Alloheim institutions with more than 50 residents in inpatient care or assisted living. Residences where the staff was not able to contribute to the study (e.g., ongoing change in management) were excluded. Individuals in ambulatory nursing care were also excluded, which accounts for more than one-third of potential study participants not being included. A total of 45 residences were visited by the study team. In those, all residents and their next of kin were informed about the study objective and methods in public presentations. Any resident, regardless of physical or mental condition, was invited to participate and was included if informed consent could be acquired. Subjects that were legally competent were included, as well as those under guardianship, provided the proband agreed to be examined and his or her legal guardian gave the written consent for study participation. Some residences also care for unusually young patients that require nursing care because of neurological disorders. For the sake of a more homogeneous study population, we decided to exclude individuals younger than 60 years for the final analysis.

### 2.3. Variables of Interest

The examinations were carried out between December 2014 and May 2016. Each residence was visited by a team consisting of a physician, two medical students and a nurse. Any proband’s demographical data and cardiovascular risk factors were assessed. Previous diagnoses and the daily medication at baseline were taken from the patient file. A physical examination included the palpation of pulses on both legs and a measurement of the ankle-brachial index. Examiners had a minimum of one month’s training in the Department of Vascular Surgery at the University Hospital of Cologne. 

**Figure 2:**
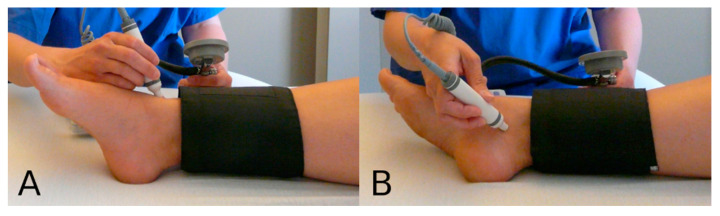
Ankle pressure measurement with a Doppler probe: anterior tibial (**A**) and posterior tibial (**B**) arteries.

The ABI measurement and calculation were performed according to the recommendations of the American Heart Association (AHA) [9]. Before the examination, the probands had a minimum of five minutes of rest in a supine position. For each leg, the anterior and posterior tibial artery systolic blood pressures were measured (Figure 2) with a sphygmomanometer and a handheld Doppler device (model dopplex d900, Huntleigh Healthcare, Cardiff, UK) with an 8 Mhz probe. Both brachial pressures were also obtained via the Doppler method. ABI was calculated by dividing the higher value of a leg’s anterior and posterior tibial artery pressures by the higher of the two brachial pressures. An ABI ≤0.9 and >1.4 were regarded as pathological.

### 2.4. End Points

We contacted the residences at regular intervals and inquired which study participants were still living there. If they had died or moved out, the date of death or censoring was noted. Furthermore, we had access to the medical reports if the patient had been treated in a hospital since the examination. An evaluation of the mortality and cardiovascular and cerebrovascular events during the first year of observation was published in 2019 [8]. Now, we report the results of an additional mortality assessment five years after the examination. We had access to data that were not available at the time of the first follow-up, leading to a slightly different patient population. To eliminate rounding errors, all ABI calculations were repeated based on the raw data of the measurements. Therefore, updated patient characteristics and PAD prevalence are given here again for completeness. 

### 2.5. Statistical Analysis

The statistical analysis was performed with IBM SPSS Statistics, version 28. A test of normality was performed for all numeric data before the statistical comparison. The data are reported as median and inter-quartile ranges (IQRs) for continuous variables and as percentages (of valid datasets) for nominal variables. For statistical comparisons of non-parametric data, the Mann–Whitney test and the Kruskal–Wallis test for independent samples were used. A chi-square test was used to compare the categorical variables. A value of *p* < 0.05 was considered to be statistically significant. Survival plots were generated using the Kaplan–Meier method. A log-rank test was used to compare the survival time between the groups defined by ABI values. To identify influencing factors on mortality, a backward stepwise multivariate Cox regression analysis was performed. In mortality analyses, only those individuals with a full five-year follow-up were included. In survival time analyses, all datasets were included.

## 3. Results

A total of 1333 participants were eligible for further analysis. A total of 1150 (86.3%) of them had a follow-up of at least five years. A total of 40 subjects (3.0%) had no follow-up, and 148 (11.1%) had a follow-up shorter than five years. The major reason for incomplete follow-up was the closing of two residences during the observation period and a software change in another with the subsequent loss of all older patient files.

### 3.1. Patient Characteristics

The median age of the population was 84 years (IQR 78–89 years). The majority (70.5%) of the participants were female. Merely 221 probands (16.6%) were mobile without assistance. More than half of the population (54.5%) were overweight or obese.

Measurement of the ankle-brachial index revealed pathological values in 739/1333 individuals (55.5% of the population). A low ABI on at least one leg (lower than or equal to 0.9) was found in 531 probands (39.9%), and a high ABI (greater than 1.4) in 236 (17.7%). A total of 449 probands (33.7% of the population) had a low ABI but no previously noted diagnosis of PAD.

To analyze the impact of pathological ABI values on mortality, we defined three groups: 592 probands (44.4% of the population) with a normal ABI on both legs, 503 (37.7%) with a low ABI on at least one leg, and 208 (15.6%) with a high ABI. The 28 probands (2.1% of the population) with a low ABI value on one leg and a high ABI on the other leg were excluded from the analyses comparing three groups. The age and sex distributions of the three groups did not differ significantly (see Table 1 for detailed patient characteristics).

### 3.2. Ankle-Brachial Index and Mortality

**Figure 3:**
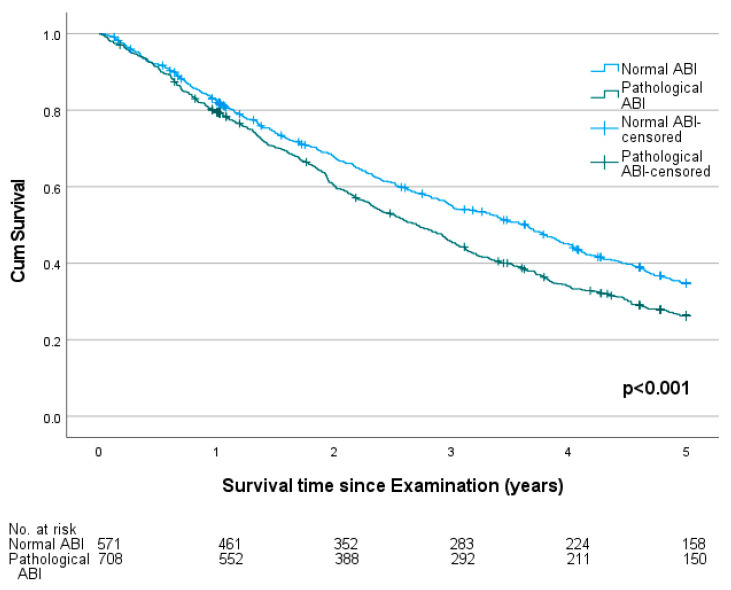
Survival plot of probands with normal (value 0.9–1.4) and pathological ankle-brachial indexes (ABIs) in a cohort of 1333 German nursing home residents 60 years and older.

**Figure 4:**
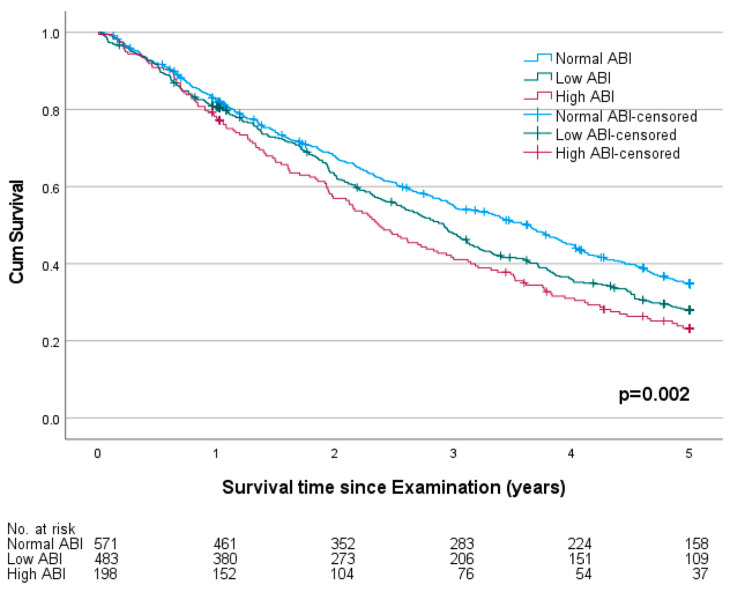
Survival plot of probands with normal, low (≤0.9) and high (>1.4) ankle-brachial indexes (ABIs) in a cohort of 1333 German nursing home residents 60 years and older.

**Figure 5:**
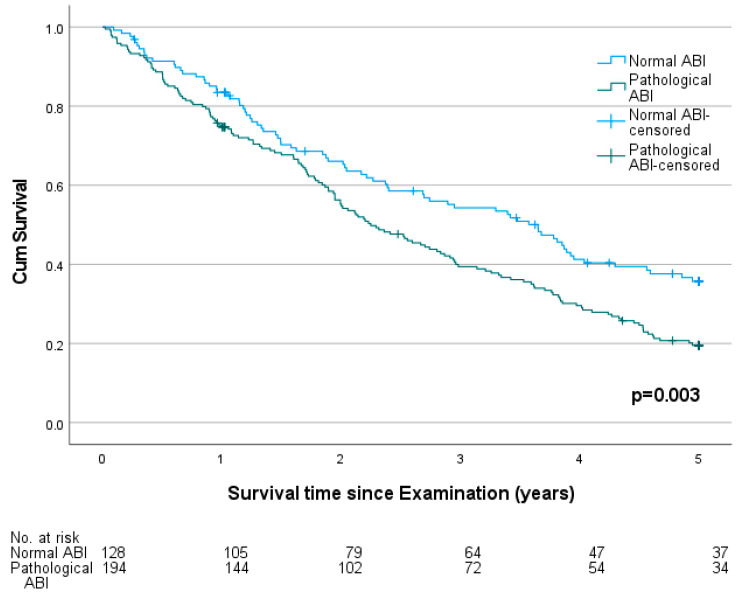
Survival plot of probands with normal (value 0.9–1.4) and pathological ankle-brachial indexes (ABIs) among the 322 probands with a preexisting diagnosis of coronary artery disease in a cohort of 1333 German nursing home residents 60 years and older.

Throughout the observation period, 835 deaths were recorded. The five-year mortality was 73.1%. The median survival time was shorter for males (2.8 years) and longer for female probands (3.1 years, *p* = 0.036). The five-year mortality was higher in probands with a pathological ABI (76.5%) compared to those with a normal ABI (68.7%, *p* = 0.003). The survival plot is shown in Figure 3. Comparing three groups of normal, low and high ABI, the mortality was significantly higher in probands with a high ABI (79.4%) than in those with a low (74.7%) or normal ABI (68.7%, *p* = 0.011). The survival plot is shown in Figure 4. In patients with a preexisting diagnosis of coronary artery disease (CAD, *n* = 322), a pathological ABI was still significantly associated with higher mortality (81.7% vs. 68.6%, *p* = 0.009). The survival plot is shown in Figure 5.

### 3.3. Ankle-Brachial Index, Patient History and Mortality

Apart from age and sex, the ABI values and several pre-existing conditions were associated with higher mortality. A backward stepwise multivariate Cox regression identified the factors listed in Table 2. Both low and high ABI were important risk factors for mortality after correction for all other recorded factors. While an abnormally high ABI was more common in individuals with diabetes mellitus (23.3%) than in those without diabetes (15.4%, *p* < 0.001), the diagnosis of diabetes mellitus alone did not constitute a significant risk factor for mortality. A high ABI was also more often found in individuals with renal insufficiency (26.4%) than in those without (15.3%, *p* < 0.001). Patients with a diagnosis of hypertension had higher mortality (74.5%) than those without (67.4%, *p* = 0.028). After correction for other factors, hypertension did not appear to be an important risk factor in the Cox regression analysis.

### 3.4. Medication and Mortality

The daily intake of statins and ACE inhibitors (as noted at baseline) was associated with a longer survival time. Therapy with diuretics was associated with a shorter survival time. Concerning other antihypertensive drugs, oral anticoagulation or platelet inhibitors, there were no significant differences in survival time. Analysis of the “pathological ABI”, “low ABI” and “known CAD” subgroups showed the results listed in Table 3.

## 4. Discussion

### 4.1. Prevalence of Peripheral Arterial Disease

To the best of our knowledge, the Allo-Study is the first study performing an ABI screening of nursing home residents in Germany. In our study cohort, the prevalence of PAD (39.9% with low ABI) was substantially higher than in the general public. A large analysis covering 87% of the German population 2009–2018 states a mean prevalence of 2.51%, with a maximum of 19% in the 75–79 year age group [2]. The getABI (German epidemiological trial on ankle-brachial index) study [3], aimed at determining the PAD prevalence in primary care patients older than 65 years, reports 19.8%. It is not surprising that nursing home residents have higher morbidity than the general public. Our study cohort was, on average, older (mean age 83.0 years) than the one examined in the getABI study (mean age 72.5 years). Furthermore, the getABI study excluded legally incompetent patients and those with a life expectancy shorter than six months. The Allo-Study recruited at the site of the nursing homes. Therefore, we could acquire informed consent from residents (or their guardians) who are usually not included in clinical studies because of limited mobility or cognitive impairment. This Italian analysis [10] of nursing home residents found a 28% prevalence of low ABI in a cohort with a mean age of 82.1 years, which is closer to our findings. 

### 4.2. Underdiagnosis of Peripheral Arterial Disease

Existing studies report an alarmingly high rate of underdiagnosis of PAD. In the PARTNERS study (Peripheral Arterial Disease Awareness, Risk, and Treatment: New Resources for Survival), more than 50% of the PAD diagnoses in a risk population were newly established [11]. A considerably high number of PAD patients do not present typical symptoms (less than 10% with classical Rose claudication in the PARTNERS study), leading to a delayed diagnosis and therapy even in patients without significant comorbidities. In our cohort, 39.9% of the population were found to have an ABI ≤ 0.9, suggesting PAD. But 84.7% of them had no PAD diagnosis, according to their patient file. In total, 33.7% of the entire study population suffered from PAD without being previously diagnosed. This indicates an even lower awareness of PAD in our cohort, possibly aggravated by reduced diagnostics in old and multimorbid patients. Frail patients and those with dementia are less likely to report (or even experience) intermittent claudication than younger, healthier individuals, and, unfortunately, even the presence of gangrene does not always prompt vascular imaging [12]. An ABI measurement can be obtained regardless of the patient’s mental and physical condition. Therefore, it can provide useful information on the extent of atherosclerotic disease even under circumstances that make patient history and clinical examination unreliable.

### 4.3. Ankle-Brachial Index and Mortality

A pathological ABI was associated with a reduced life expectancy. The morbidity and mortality following a diagnosis of peripheral arterial disease have been shown to be higher than those after a myocardial infarction [13]. Furthermore, there is a known association between PAD and CAD. In the PARTNERS Study [11], 16% of a risk population presented with both PAD and CAD; angiographic examinations found CAD in up to 62% of symptomatic PAD patients [14]. It is conceivable that the pathological ABI value is merely an indicator for underlying cardiac disease. The detection of CAD in elderly and multimorbid patients can be impaired by the same mechanisms that lead to an underdiagnosis of PAD. Therefore, the actual prevalence of CAD in the study group could have been higher than reported. Nevertheless, we observed a higher mortality with pathological ABI even in the subgroup of patients with a known CAD and after correction for comorbidities in the Cox regression analysis. 

The finding of an abnormally high ABI was significantly associated with higher mortality. This is consistent with previous research [15], although some analyses examining ABI excluded subjects with values greater than 1.4 or have inconclusive results [16]. A high ABI tends to be attributed to medial arterial calcification (MAC), although the pathophysiology is still subject to research. Apparently, “high ABI” and “MAC” cannot be used synonymously [17]. As MAC can result in incompressible arteries, lumen stenoses are less likely to be detected via ABI measurement. Therefore, the “high ABI” subgroup might have contained a significant portion of PAD patients. Existing research agrees on a strong association of diabetes mellitus and abnormally high ABI [18]. In our study cohort, a high ABI was more common in diabetic probands, but a significant impact on mortality was only seen for high ABI, not diabetes. A high ABI is also discussed to be associated with end stage renal disease. Renal disease was common among our probands, and it was an important risk factor for mortality in itself. This might explain to some extent why high ABI was associated with higher mortality. Nevertheless, this effect was visible even after correction for all known previous diagnoses of our probands, including diabetes and renal disease.

The Cox regression suggests an important role of the ABI as a risk factor for mortality independent of numerous underlying conditions. Two existing analyses of nursing home residents did not confirm a statistically significant impact of ABI on mortality [10,19], possibly because of smaller sample size and shorter observation periods. The role of a low ABI in the prediction of cardiovascular mortality has already been extensively evaluated, e.g., in the 2008 meta-analysis of the Ankle Brachial Index Collaboration [6] and this more recent meta-analysis [20]. The vast majority of the studies included in those analyses had substantially younger cohorts than the Allo-Study. So while our key findings are not new, they complete the existing research by adding data for older patients. 

### 4.4. Medication and Mortality

Undertreatment of PAD patients was observed in our cohort, concurring to previous research [2,11]. Therapy with statins at baseline was associated with a reduced overall mortality, as observed in larger trials [21]. Current guidelines by the European Society of Cardiology (ESC) and the European Society for Vascular Surgery (ESVS) [22], the European Society of Vascular Medicine (ESVM) [23,24] and the American College of Cardiology and American Heart Association (ACC/AHA) [25] agree on the recommendation of a lipid-lowering therapy for all patients with PAD. However, merely one-third (32.6%) of the patients with a known PAD diagnosis were receiving it. We did not observe a significant impact of platelet inhibitors or anticoagulants on mortality, neither in the whole population nor in the PAD and CAD subgroups. Evidence for the benefit of aspirin for PAD patients is not as clear as it is for CAD, namely because of fewer and smaller randomized trials with PAD patients [26]. A randomized trial addressing asymptomatic individuals selected for a low ABI failed to show the benefit of aspirin for this population [27]. Recent research in favor of aspirin plus rivaroxaban [28,29] was not yet available at the time of our examinations. Merely 2.4% of our study population were receiving any type of oral anticoagulant plus aspirin. Since the particular substance was not recorded, we cannot draw any conclusions from the outcomes of those probands. Treatment with platelet inhibitors or anticoagulants is limited in elderly and multimorbid patients because of concerns for a higher rate of bleeding complications [30,31], which may lessen its net benefit. Nevertheless, the possibility of PAD should not be overlooked in these patients to allow the best medical treatment for each individual.

### 4.5. ABI Screening

The mortality analysis suggests that ABI is useful to identify patients with a high risk for cardiovascular mortality. Although the evidence supporting an ABI screening in the general public is still considered incomplete by European [32] and US experts [33], both the European Society of Cardiology [22] and the American Heart Association [25] recommend ABI measurements in asymptomatic individuals above an age threshold or with risk factors. The Danish Viborg Vascular (VIVA) randomized controlled trial showed a promising reduction of mortality by 7% through a combined screening for abdominal aortic aneurysm, PAD and hypertension in men 65–74 years of age [34]. Screening appears to be most useful in a population with a high prevalence and inadequate diagnostics and therapy. However, in old and very old patients, measures of secondary prevention cannot be expected to be effective in the short term and may not be appropriate in end-of-life care. Evaluating the potential of an ABI screening in this vulnerable population would be subject to practical and ethical restrictions. Naturally, detection and treatment of PAD at a younger age and in earlier stages would be preferable to improve the patient’s long-term prognosis.

The alarming rate of underdiagnosis suggests care for elderly patients with PAD might be improved by easier access to ABI measurements. PAD complications can result in severe loss of function and quality of life [1] and can aggravate elderly patients’ need for support, with the consequence of an additional economic burden. Therefore, serious clinical signs of PAD, such as rest pain or chronic ulcers, should not be overlooked because of the patient’s age. The 2019 ESVM guideline recommends routine pulse examinations in elderly patients as they may aid in preventing PAD complications [23]. Therapeutic decisions should be individualized and, as the risk of iatrogenic complications grows with age, life expectancy and personal fitness should be taken into account. In those patients who have the potential to profit from therapy, a timely diagnosis of PAD is important as it improves the prognosis [35]. Its simplicity makes the ABI a useful first-line diagnostic tool.

### 4.6. Limitations

Obtaining an accurate ABI measurement can be impaired in the presence of edema or obesity and might not be possible in the case of a painful leg ulcer. Nevertheless, we could acquire ABI values for 99% of limbs, the most common reason for missing values being lower extremity amputation. To reduce inter-observer differences, the study teams received identical training at the University Hospital of Cologne for at least one month prior to the examinations on site. 

Incompressible arteries of the lower leg can lead to a higher ABI, masking a low perfusion pressure of the limb. In clinical practice, especially in diabetic patients and those with nephropathy, additional methods such as toe brachial index or Doppler waveform analysis [36] are applied for a more reliable assessment. At the time of enrollment, the necessary devices were not at our disposal. Nevertheless, our observations suggest a high ABI, although not reliably confirming or ruling out PAD, bears prognostic information in itself.

The proband’s daily medication was only recorded at baseline and may have changed throughout the observation period, limiting conclusions on medication effects.

A five-year follow-up concerning mortality was completed in 86.3% of subjects. The mortality analysis was planned to be complemented by a count of cardiovascular events (CV events). Unfortunately, we were not provided with all the necessary documents to continue CV event recording after the one-year follow-up, predominantly in the case of deceased participants. To avoid a biased presentation, we did not conduct any statistical analyses of CV events for the five-year follow-up.

## 5. Conclusions

The five-year observation of our cohort of 1333 German nursing home residents confirmed an association between pathological ABI values and mortality. Considering that nursing home residents have been shown to have a priori a very short life expectancy [37], it is remarkable that this observation can be made as clear as in other patient groups.

One in three nursing home residents was found to suffer from undiagnosed PAD. In geriatric patients nearing the end of life, caregivers may have deliberately made a decision to limit diagnostic and therapeutic measures. However, in patients with life expectancy and fitness that justify those measures, easier access to ABI measurements has the potential to aid in preventing or alleviating PAD complications.

## Figures and Tables

**Table 1:** Patient characteristics, diagnoses and daily medication at baseline in a cohort of 1333 German nursing home residents 60 years and older.

		Total (*n* = 1333)	Normal ABI (*n* = 592)	Low ABI (*n* = 503)	High ABI (*n* = 208)	*p*
Age—y		84 (78–89)	84 (78–89)	84 (77–89)	85.5 (79–89.75)	0.137 #
sex	male	393 (29.5)	173 (29.2)	137 (27.2)	72 (34.6)	0.14
	female	940 (70.5)	419 (70.8)	366 (72.8)	136 (65.4)	
Tobacco use		573 (43.9)	241 (41.3)	240 (48.8)	83 (41.3)	0.033 *
Coronary artery disease		338 (25.4)	136 (23.0)	135 (26.8)	58 (27.9)	0.22
Peripheral vascular disease (previously known)		138 (10.4)	33 (5.6)	78 (15.5)	22 (10.6)	<0.001 *
Carotid disease		79 (6.0)	31 (5.2)	34 (6.9)	13 (6.3)	0.52
Hypertension		1044 (78.4)	428 (72.4)	417 (82.9)	173 (83.2)	<0.001 *
Dyslipidemia		382 (28.7)	176 (29.7)	143 (28.5)	57 (27.4)	0.79
Diabetes mellitus		401 (30.1)	147 (24.8)	160 (31.9)	81 (38.9)	<0.001 *
Renal insufficiency		305 (22.9)	99 (16.7)	127 (25.3)	68 (32.7)	<0.001 *
Cardiac arrhythmia		390 (29.3)	165 (27.9)	132 (26.4)	83 (39.9)	<0.001 *
Chronic heart failure		281 (21.1)	109 (18.4)	113 (22.5)	53 (25.5)	0.063
Ischaemic stroke		297 (22.4)	107 (18.1)	127 (25.4)	57 (27.4)	0.003 *
Cognitive dysfunction		643 (48.2)	295 (49.8)	237 (47.1)	98 (47.1)	0.62
COPD		150 (11.3)	55 (9.3)	66 (13.1)	28 (13.5)	0.083
Cancer		219 (16.4)	104 (17.6)	67 (13.3)	41 (19.7)	0.058
Aspirin		481 (37.8)	193 (33.3)	216 (43.8)	72 (36.2)	0.002 *
Clopidogrel		49 (3.9)	16 (2.8)	24 (4.9)	9 (4.5)	0.17
Oral anticoagulants		206 (15.8)	98 (16.6)	61 (12.1)	47 (22.6)	0.002 *
Beta blockers		594 (45.6)	245 (41.4)	239 (47.5)	110 (52.9)	0.009 *
ACE inhibitors		659 (50.6)	276 (46.6)	277 (55.1)	106 (51.0)	0.020 *
Calcium channel blockers		291 (22.3)	128 (21.6)	122 (24.3)	41 (19.7)	0.36
Diuretics		733 (56.3)	311 (52.6)	296 (58.8)	126 (60.6)	0.047 *
Statins		358 (27.5)	174 (29.4)	129 (25.6)	55 (26.4)	0.36
Oral antidiabetics		145 (11.1)	51 (8.6)	72 (14.3)	22 (10.6)	0.011 *
NASIDs		130 (10.0)	58 (9.8)	45 (8.9)	27 (13.0)	0.26
Metamizole		511 (39.2)	223 (37.7)	202 (40.2)	86 (41.3)	0.56
Opioid		247 (19.0)	104 (17.6)	108 (21.5)	35 (16.8)	0.18
Psychotropic medications		762 (58.6)	345 (58.3)	290 (57.8)	127 (61.4)	0.67

Age is reported as median (inter-quartile range), and all other values are reported as *n* (%); ABI: ankle-brachial index, COPD: chronic obstructive pulmonary disease, NSAID: non-steroidal anti-inflammatory drug; #: The group’s age distribution was compared by an Independent samples Kruskal–Wallis test. *: The chi-square statistic used for all categorical variables is significant at the 0.05 level.

**Table 2:** The most important influencing factors on the survival time in a five-year observation of a cohort of 1333 German nursing home residents 60 years and older, according to Cox regression analysis.

	Hazard Ratio	95.0% Confidence Interval of the Hazard Ratio	*p*
Age	1.045	1.04–1.06	<0.001
Female sex	0.764	0.65–0.9	0.001
ABI category			0.031
− low ABI	1.205	1.03–1.41	0.021
− high ABI	1.238	1.01–1.52	0.040
Renal insufficiency	1.322	1.12–1.56	<0.001
Ischaemic stroke	1.160	0.98–1.38	0.087
Cognitive dysfunction	1.170	1.01–1.35	0.032
COPD	1.537	1.25–1.89	<0.001
Cancer	1.295	1.08–1.56	0.006

ABI: ankle-brachial index, COPD: chronic obstructive pulmonary disease; the following factors were also included into the analysis, but were not shown to have a significant impact: coronary artery disease, carotid disease, tobacco use, hypertension, dyslipidemia, diabetes mellitus, cardiac arrhythmia, chronic heart failure.

**Table 3:** Association of daily medication at baseline and survival time in a five-year observation of a cohort of 1333 German nursing home residents 60 years and older.

	Study Cohort (*n* = 1333)	Pathological ABI Subgroup (*n* = 729)	Low ABI Subgroup (*n* = 503)	Known CAD Subgroup (*n* = 322)
Statins	Longer survival, *p* = 0.001	Longer survival, *p* = 0.002	Longer survival, *p* = 0.006	Longer survival, *p* = 0.007
Diuretics	Shorter survival, *p* = 0.002	Shorter survival, *p* = 0.027	(*p* = 0.075)	Shorter survival, *p* = 0.045
ACE inhibitors	Longer survival, *p* = 0.024	(*p* = 0.05)	(*p* = 0.55)	(*p* = 0.10)
Metamizol	(*p* = 0.075)	Shorter survival, *p* = 0.036	(*p* = 0.10)	(*p* = 0.78)
Nonsteroidal anti-inflammatory drugs	(*p* = 0.19)	(*p* = 0.277)	(*p* = 0.42)	Shorter survival, *p* = 0.026
Psychotropic medications	(*p* = 0.066)	(*p* = 0.57)	(*p* = 0.068)	Shorter survival, *p* = 0.018
No statistically significant difference in survival time in all three groups: Aspirin, Clopidogrel, Oral anticoagulation, Beta blockers, Calcium channel blockers, Opioids, Oral antidiabetics				

ABI: ankle-brachial index, CAD: coronary artery disease, ACE: angiotensin converting enzyme.

## Data Availability

The data presented in this study are available on request from the corresponding author. The data are not publicly available due to privacy restrictions.
